# Femur Shaft Fracture in Newborns: A Report of Two Cases

**DOI:** 10.7759/cureus.12504

**Published:** 2021-01-05

**Authors:** Kishore Vellingiri, Sagar Venkataraman, Arun H Shanthappa, Hariprasad Seenappa

**Affiliations:** 1 Orthopaedics, Sri Devaraj Urs Academy of Higher Education and Research, Kolar, IND

**Keywords:** breech presentation, caesarean section, femur fracture

## Abstract

Birth injuries caused by trauma during the childbirth process are very rare. They are a cause of significant neonatal morbidity despite improved obstetric and perinatal care, particularly in developing countries. Our current research, consisting of two newborns with a femur fracture, aims to shed light on their treatment strategy.

## Introduction

In previous studies, 0.02% of the long bone fracture and 1.1% of the foetal injury found in Caesarean section were reported by Alexander et al. [1]. Breech presentation, which accounts for approximately 3-4% of deliveries, is a risk factor for perinatal mortality and morbidity [2]. Femoral fractures occur after vaginal breech delivery or difficult delivery of the pelvis by the infant to the breech position and vigorous Caesarean extraction is required [3].

## Case presentation

**Figure 1:**
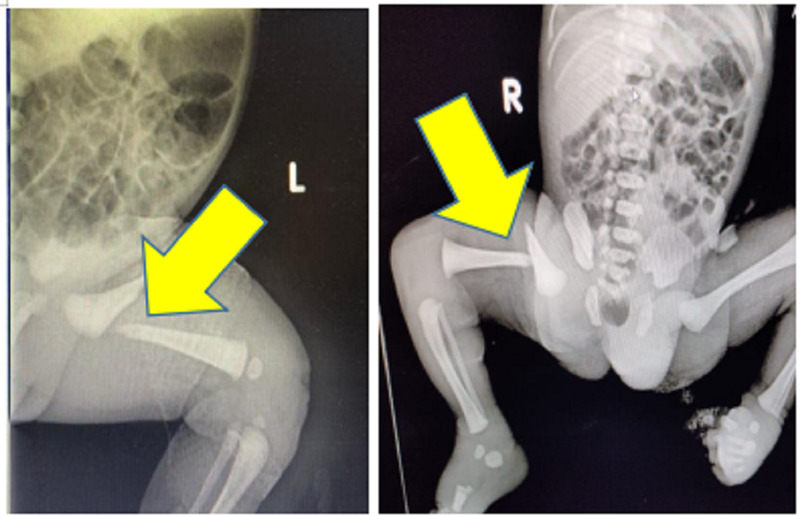
Radiographs of Baby 1 & Baby 2

Two newborn babies were delivered by Caesarean section in our tertiary care centre, Kolar, Karnataka. The first was a 36-week preterm male infant small for gestation. Baby presented with swelling and deformity noted over left thigh after difficult labor. Baby was diagnosed with a closed proximal fracture on the left side of the femur. The second was a 38-week female infant small for gestation. Following difficult labor, baby presented with swelling and deformity over the right thigh. Baby was diagnosed with a closed proximal fracture on the right side of the femur. Radiographs of the babies are shown in Figure 1.

**Figure 2:**
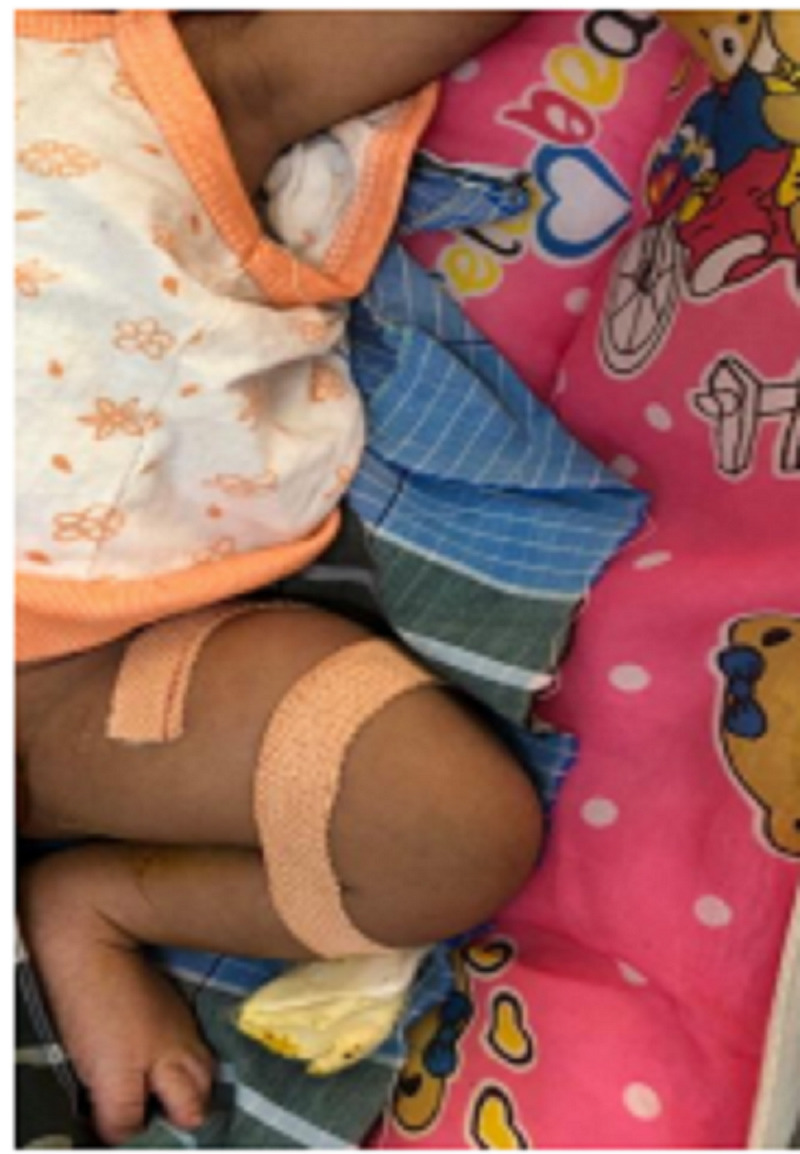
Strapping Involving Thigh and Leg

**Figure 3:**
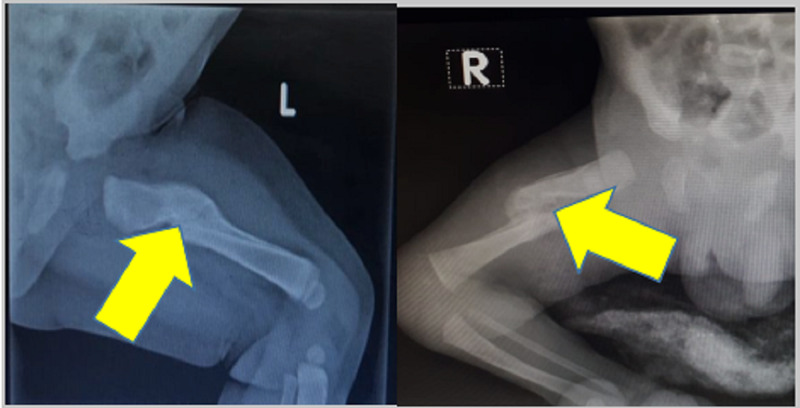
Fracture Unions of Baby 1 & Baby 2

Both newborn babies were treated conservatively by applying strapping as shown in Figure 2. Fracture union was verified by x-rays at monthly intervals of six months as shown in Figure 3 and the babies were monitored at regular intervals. Both fractures were united without any complications by a conservative procedure.

## Discussion

In newborns following Caesarean section, fractures themselves are usually located in the shaft of the femur and are of spiral nature [4]. The predisposing factors are low or high birth weight, advanced age of the mother, preterm delivery, and uterine fibrinomas [5]. Emergency Caesarean delivery carries a higher risk of long bone fracture than vaginal delivery. Prematurity, mal-presentation, abnormal lie, and multiple pregnancy can predispose to long bone fractures. The prognosis of birth-associated long bone fractures is good [6].

Swelling of the leg, lack of motion, and discomfort when changing diapers are the most common signs [7]. Neonates delivered by Caesarean section in the breech presentation should be closely monitored after birth [8]. Fracture diagnosis is typically based on a clinical review. The key characteristics are swelling and decreased strength of broken limbs and extreme pain in passive movements. Fractures can sometimes be identified inadvertently during the x-ray investigation for other causes, such as respiratory distress [9]. There are many approaches available for the treatment of femoral neonatal fractures. Only the separation of distal femoral epiphysis can require intervention in the event of instability and extreme epiphysis displacement. The use of Pavlik brace in infants with femoral fractures is considered to be easier to apply, avoiding skin irritation [10,11]. The use of Pavlik harness has less traumatic skin irritation than other methods such as Bryant traction. The correlation between vaginal and breech delivery still has the propensity to femur fracture since excess force is needed [12]. Remind the surgeon that any forceful extraction can result in long bone injuries. Care should also be taken before and after delivery to rule out injuries [13].

In order to prevent such inadvertent complications, the surgeon should be very careful during the delivery of the infant, avoid energetic traction, and prepare his incisions. Sufficient relaxation of the uterus needs to be achieved. Clavicles and other long bones should be palpated after a difficult delivery. However it is important to remember that long bone fractures in children recover quickly without significant intervention [14]. Caesarean delivery does not minimize the risk of birth trauma associated with femoral fractures and there is a risk of femoral fracture in case of emergency Caesarean section. Premature neonates with intrauterine growth retardation and long-term overall parenteral nutrition should be supplemented with calcium, phosphorus, and vitamin D in order to overcome osteopenia of prematurity [15].

## Conclusions

Both fractures in newborn babies were treated conservatively by strapping. Both fractures were united without any complications by a conservative procedure.

Proper delivery protocol includes urgent examination. It is appropriate to carry out orthopedic consultation of the newborn in the midst of forced obstetric maneuvers.

## Appendices

This case study was presented as E-poster presentation at the 19th Annual Conference of Orthopaedic Association of South Indian States (OASISCON 2020) Virtual Conference held on 27th to 29th November 2020. The abstract of this article is published in the online supplement of the conference journal.
